# Máncel Enrique Martínez Durán 1953-2019

**Published:** 2019-12-30

**Authors:** Fernando De la Hoz

**Affiliations:** 1 Departamento de Salud Pública, Universidad Nacional de Colombia, Bogotá, D.C., Colombia Universidad Nacional de Colombia Departamento de Salud Pública Universidad Nacional de Colombia BogotáD.C Colombia

Es difícil darle un adiós definitivo a aquellas personas que queremos y respetamos, ya sea por sus cualidades personales o porque hemos crecido -biológica, profesional o intelectualmente- junto a ellos, o porque han sido nuestros mentores en algún periodo de nuestras vidas.

Decirle adiós a Máncel Enrique Martínez Durán representa para mí un reto aún más difícil, porque yo llegué a admirar sus talentos políticos e intelectuales; le tomé un gran cariño porque crecí intelectual y profesionalmente a su lado y, además, fue mi mentor por, al menos, 15 años.

Se agolpan en mi memoria todos los recuerdos que tengo de él, recuerdos adquiridos en una etapa temprana de mi vida profesional en la Salud Pública, donde Máncel se encargó de enseñarme el ABC de la epidemiología práctica y de alto impacto en salud pública que él practicaba con éxito en el Instituto Nacional de Salud.

La mayoría de esos recuerdos son alegres, siempre lo imagino cantando vallenatos y ensayando algún pase de baile, algo que relajaba la tensión en los momentos difíciles de nuestro trabajo conjunto, que no eran pocos dado el volumen de trabajo urgente que debíamos procesar debido a la importancia nacional e internacional que, gracias a su empuje y a su visión, iba adquiriendo el pequeño grupo de trabajo en vigilancia epidemiológica que él lideraba.

Pero, también, algunos tristes como cuando renunció al Instituto Nacional de Salud por diferencias con la Dirección de turno y se marchó a montar una especialidad en Epidemiología en la Escuela de Medicina Juan N. Corpas. Máncel nació en la ciudad de Valledupar en 1953, estudió Medicina en la Universidad Nacional de Colombia de donde se graduó en 1977, y se vinculó al Instituto Nacional de Salud en 1979. Fue atraído a esa institución por el Dr. Álvaro Aguilera, veterano epidemiólogo del Instituto y experto en fiebre amarilla, a quien Máncel impresionó con su capacidad de trabajo e inteligencia durante las actividades de control del brote de fiebre amarilla en la Sierra Nevada de Santa Marta entre 1978 y 1979, mientras era médico rural. Posteriormente, se graduó de magíster en Salud Pública en la Universidad de Antioquia con una tesis sobre la epidemiología de la exposición y el manejo de la rabia humana en Colombia, lo que lo convirtió en referente nacional e internacional para las actividades de control de la rabia durante la década de 1980.

Sus aportes a la salud pública nacional e internacional son numerosos - estudios sobre rabia, fiebre amarilla, dengue y dengue hemorrágico, como se llamaba entonces- pero voy a citar solo cuatro que, a mi juicio, resumen gran parte de su visión y su capacidad de gestión. Tres de estos hitos se dieron durante su carrera en el Instituto Nacional de Salud y uno durante su etapa como profesor de Epidemiología en la Escuela de Medicina de la Fundación Universitaria Juan N. Corpas.

Lo primero que destaco durante su carrera en el Instituto, fueron sus aportes al conocimiento sobre la epidemiología de la hepatitis B y delta en Colombia, al conocimiento de las poblaciones altamente endémicas para estos virus y el establecimiento de un programa de vacunación contra la hepatitis B que ha salvado miles de vidas. Ese programa de vacunación que él inició en las localidades rurales de Ciénaga (Magdalena) con unos pocos fondos provistos por la Organización Panamericana de la Salud (OPS) y los *Centers for Disease Control and Prevention* (CDC), creció hasta convertirse en un programa nacional de vacunación que luego fue copiado por todos los países de Latinoamérica y, está a punto de lograr que Colombia sea declarado ‘país libre de la transmisión vertical de la hepatitis B’. Lo bueno de ejercer la salud pública es que los beneficios de un trabajo incansable como el de Máncel (trabajaba sábados y domingos sin quejarse), alcanzan a mucha más gente de la que uno nunca ha soñado, y lo paradójico es que, al ser transformado en políticas públicas, después de unos años nadie recuerda cómo y quien empezó todo.

Otro hito dentro de su carrera en el Instituto fue su participación en en las pandemias de cólera de los años 80 y 90 que tocaron a Colombia entre 1991 y 1995. Pese a que nunca había visto un caso de cólera ni había trabajado previamente en su control o prevención, Máncel fue capaz de preparar a su pequeño equipo de trabajo y convertirlo en un grupo indispensable en la respuesta que el país le dio a esta amenaza. Muchas fueron las investigaciones de campo de brotes de cólera que hicimos bajo su liderazgo y muchas las publicaciones que el grupo realizó, incluyendo la primera descripción clínica de la enfermedad en Colombia después de más de 100 años y la primera descripción de los factores de riesgo para su transmisión. Además de su participación en el estudio de la epidemiología, la prevención y el control del cólera, también colideró la iniciativa para probar una nueva vacuna oral contra el cólera que había sido probada anteriormente en Bangladesh y que, aunque no necesitó ser implementada en Colombia, sí demostró que podía inducir anticuerpos protectores en personas sin experiencia inmunológica previa contra ese agente.

Pero, sin ninguna duda, su logro más grande en el Instituto Nacional de Salud fue la creación del Programa de Epidemiología de Campo. Máncel aceptó el reto propuesto por el director del Instituto de la época (1992), doctor Antonio Iglesias Gamarra, de implementar un programa de formación de epidemiólogos de campo que suplieran con prestancia la demanda de personas entrenadas para ejercer la labor de vigilancia e investigación epidemiológica en el nivel local, departamental y nacional.

Superando muchos obstáculos de todo tipo, Máncel lideró la implementación del Programa, que inicialmente se llamó Programa Aplicado de Entrenamiento en Epidemiología de Campo (PAEEA), en un esquema que, para la época, era *sui generis* ya que los residentes en formación no recibían un sueldo del CDC, como había sido usualmente la costumbre en otros programas alrededor del mundo. Esta característica ha logrado, creo yo, que este importante legado se sostenga hasta hoy cuando 120 personas se han capacitado como epidemiólogos de campo y más de 2.000 han recibido algún tipo de capacitación en epidemiología aplicada, básica o avanzada. El Programa de Formación en Epidemiología de Campo ha intervenido en un sinnúmero de problemas de salud pública del país, como las investigaciones de campo en encefalitis equina venezolana, zika, chikungunya, cólera, intoxicaciones y desastres naturales como el terremoto en el Eje Cafetero, etc.

Posteriormente, Máncel se instaló en la Escuela de Medicina de la Fundación Universitaria Juan N, Corpas, donde colideró la creación de una escuela de formación a nivel de especialidad en Epidemiología que cuenta con varias docenas de graduados. Este programa llevó la formación en Epidemiología a muchas ciudades del país en departamentos que carecían de ese recurso y, así, proveyó la oportunidad a muchos médicos y otros profesionales de la salud de capacitarse en Epidemiología sin tener que alejarse mucho de su lugar de práctica profesional.

Los versículos 4 a 6 del capítulo 11 del libro del Eclesiastés dicen: “El que al viento observa, no sembrará; y el que mira a las nubes, no segará”. Máncel era la clase de persona que no se cohibía por los problemas que su interés por la salud pública podrían acarrearle, sino que empezaba a actuar en la dirección que él creía era la correcta para resolver un problema, sin reparar en los obstáculos y sin oír mucho a aquellas voces que querían desanimarlo.

Hoy le decimos adiós a su entusiasmo por el trabajo de campo en Epidemiología, por enseñar a otros, y a su pasión por la Salud Pública, pero su recuerdo seguirá vivo por mucho tiempo entre todos los que tuvimos el privilegio de conocerlo de cerca, de apreciar sus virtudes y fortalezas, y de compartir el trabajo diario con él.

Paz en su última morada.

